# Perceptual Pattern of Cleft-Related Speech: A Task-fMRI Study on Typical Mandarin-Speaking Adults

**DOI:** 10.3390/brainsci13111506

**Published:** 2023-10-25

**Authors:** Yun Bai, Shaowei Liu, Mengxian Zhu, Binbing Wang, Sheng Li, Liping Meng, Xinghui Shi, Fei Chen, Hongbing Jiang, Chenghui Jiang

**Affiliations:** 1 Department of Oral and Maxillofacial Surgery, The Affiliated Stomatological Hospital of Nanjing Medical University, Nanjing 210029, China; baiyun@stu.njmu.edu.cn (Y.B.);; 2 Jiangsu Province Key Laboratory of Oral Diseases, Nanjing 210029, China; 3 Jiangsu Province Engineering Research Center of Stomatological Translational Medicine, Nanjing 210029, China; 4 Department of Radiology, Jiangsu Province Hospital of Chinese Medicine, Affiliated Hospital of Nanjing University of Chinese Medicine, Nanjing 210004, China; 5 Department of Children’s Healthcare, Women’s Hospital of Nanjing Medical University, Nanjing Maternity and Child Health Care Hospital, Nanjing 210004, China; 6 Department of Electrical and Electronic Engineering, Southern University of Science and Technology, Shenzhen 518055, China

**Keywords:** task-fMRI, cleft palate speech, speech perception, dual-stream model, glottal stop

## Abstract

Congenital cleft lip and palate is one of the common deformities in the craniomaxillofacial region. The current study aimed to explore the perceptual pattern of cleft-related speech produced by Mandarin-speaking patients with repaired cleft palate using the task-based functional magnetic resonance imaging (task-fMRI) technique. Three blocks of speech stimuli, including hypernasal speech, the glottal stop, and typical speech, were played to 30 typical adult listeners with no history of cleft palate speech exploration. Using a randomized block design paradigm, the participants were instructed to assess the intelligibility of the stimuli. Simultaneously, fMRI data were collected. Brain activation was compared among the three types of speech stimuli. Results revealed that greater blood-oxygen-level-dependent (BOLD) responses to the cleft-related glottal stop than to typical speech were localized in the right fusiform gyrus and the left inferior occipital gyrus. The regions responding to the contrast between the glottal stop and cleft-related hypernasal speech were located in the right fusiform gyrus. More significant BOLD responses to hypernasal speech than to the glottal stop were localized in the left orbital part of the inferior frontal gyrus and middle temporal gyrus. More significant BOLD responses to typical speech than to the glottal stop were localized in the left inferior temporal gyrus, left superior temporal gyrus, left medial superior frontal gyrus, and right angular gyrus. Furthermore, there was no significant difference between hypernasal speech and typical speech. In conclusion, the typical listener would initiate different neural processes to perceive cleft-related speech. Our findings lay a foundation for exploring the perceptual pattern of patients with repaired cleft palate.

## 1. Introduction

Congenital cleft lip and palate (CLP) is one of the most common deformities in the craniomaxillofacial region which affects nearly one in every five hundred newborns. It can cause abnormal facial morphology and hearing, speech, and language dysfunction [1]. Therefore, the sequential treatment of those patients involves multiple disciplines, including plastic surgery, the orthodontics, speech therapy, etc. Various treatments are delivered according to the development of the patient from birth to early adulthood, in order to address either the structural or functional deformities [2]. Cleft palate speech is a series of speech disorders associated with cleft palate and velopharyngeal insufficiency [3]. According to the formation mechanism, cleft palate speech is classified into passive cleft palate speech (PCPS) and active cleft palate speech (ACPS). PCPS is directly caused by structural defects of the palate and is characterized by hypernasality, nasal emission, and inadequate oral pressure during articulation. PCPS can disappear immediately after cleft palate repair and velopharyngeal reconstruction. ACPS, also known as compensatory misarticulation, can compensate for the structural defects of the cleft palate. There are various subtypes of ACPS, which include the glottal stop, lateralized and palatalized speech, etc. [4].

The development of brain science and improvements in functional magnetic resonance imaging (fMRI) technology have provided an integrated approach to uncovering the neural mechanisms of various behaviors and diseases. Several brain structure studies on the population with CLP have reported changes, including multiple degrees of midline abnormalities, increased frontoparietal region volume, and decreased temporo-occipital and cerebellar region volume [5,6,7]. Brain studies have also identified structural alterations in functional brain regions related to socialization, such as the ventral frontal cortex [8,9]. Morphological studies on gray matter volume have revealed that the development of cleft palate speech can be related to a series of structural changes, such as reduced gray matter volume in several language-related brain regions [10]. Another study on lexical processing function reported that patients with unilateral cleft lip and palate (UCLP) had delayed and prolonged blood-oxygen-level-dependent (BOLD) responses in several brain regions. Thus, children with CLP may use different neural circuits for language processing than their peers without CLP [11]. Another study reported compensatory activation in reading task-related brain regions when processing simple tasks. However, activation during complex tasks was significantly reduced. Adult patients with nonsyndromic cleft lip palate (NSCLP) might also use a different shared neural network during reading assignments [12]. Chen’s team has systematically evaluated the influence of speech therapy on patients with NSCLP. Their findings showed that an underlying structural and functional alteration in the brain might lead to speech and language dysfunction in patients with NSCLP [13,14,15,16]. While previous studies have revealed potential associations between structural and functional brain abnormalities related to cleft palate speech, they have not fully elucidated the neural mechanisms that underlie the development of cleft-related speech.

Speech perception is the basis of speech development and production. According to the dual-stream model by Hickok and Poeppel, speech perception involves two distinctive parallel processing streams (the ventral and dorsal streams). The ventral stream connects the temporal lobe and the prefrontal cortex and primarily identifies auditory stimuli, mapping the received auditory information to conceptual representations, performing sound–meaning mapping, and understanding the perceived speech. The dorsal stream connects the posterior dorsal temporal lobe, inferior parietal sensory area, and prefrontal lobe. It comprises the speech articulation interface during speech production. It maps speech sounds to their articulatory–motor representations [17,18].

**Figure 1:**
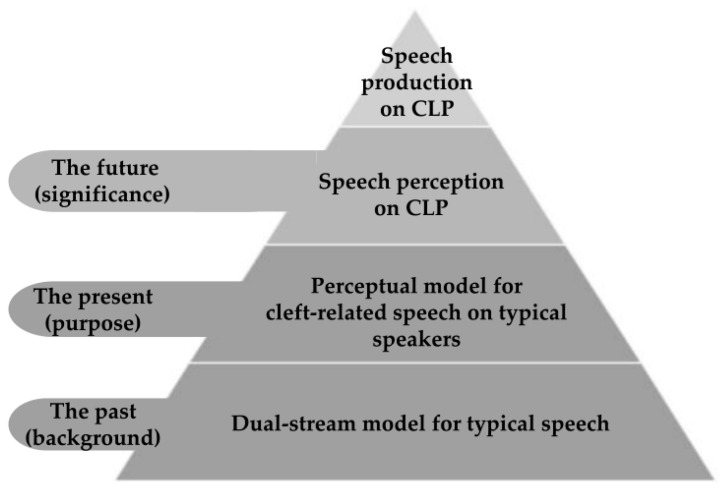
Schematic diagram of the background, purpose, and significance of the research.

How cleft-related speech is perceived is yet to be deeply explored. A behavioral study performed by our research team revealed that listeners used different spectral moments to perceive the place of consonant articulation produced by patients with cleft palate compared with those with typical speech [19]. Do the perception of typical speech and that of cleft-related speech share a similar pattern? Is the dual-stream model applicable to cleft-related speech? To investigate this question, we proposed the hypothesis that typical adult listeners may utilize a distinct neural pattern when perceiving cleft-related speech as compared to typical speech. The current study aimed to explore the perceptual pattern of cleft-related speech in typical adult speakers via task-fMRI. Our results could lay a foundation for further investigation into the speech perception and production of speakers with cleft palate (Figure 1).

## 2. Materials and Methods

### 2.1. Participants

In total, 30 typical adult volunteers (15 men and 15 women, with a mean age of 23.1 years, standard deviation = 2.36) were recruited. The inclusion criteria were as follows: (1) individuals without a history of exploration of any cleft palate speech, (2) typical listeners with normal peripheral hearing ability (a mean hearing threshold of ≤20 dB for air conduction in binaural pure-tone audiometry), (3) with normal or corrected to normal vision, (4) with right-handedness, (5) individuals whose primary language of communication is native Chinese Mandarin, (6) without congenital conditions, (7) without a history of mental or neurological dysfunction, and (8) with a Wechsler Adult Intelligence Scale (Chinese version) (Third Edition) score of >90.

The exclusion criteria were as follows: (1) individuals with palatopharyngeal structure and function or other craniomaxillofacial deformities or defects, (2) typical listeners with hearing or visual impairment, (3) with intellectual impairment, and (4) with a history of claustrophobia or contraindications to MRI.

### 2.2. Stimuli

Two speech samples, which included one cleft-related hypernasal speech (hypernasal speech for short) and one cleft-related glottal stop (glottal stop for short), were extracted from the cleft palate speech database of the research team. The speech samples in the database were recorded in a quiet recording room (with a background noise less than 30 dB). Both speech samples were articulated by male patients aged 24–48 months. The diagnostic labels in the cleft palate speech database were determined via a joint assessment conducted by two speech therapists from the cleft lip and palate treatment team. An additional speech sample was collected from a gender- and age-matched typically developed child. All speech samples were age-appropriate daily oral phrases. Thirty-two phrases were extracted from each sample. There were 96 speech stimuli (Supplementary Table S1). All samples were processed by low-pass filtering (3.8 kHz). Then, they were equalized in terms of loudness and duration using an acoustic analysis software (Praat, version 6.2). Stimulus presentation was performed using E-prime 3.0 software (Psychology Software Tools, Inc., Pittsburgh, PA, USA, version 3.0.3.60). It was loaded on a brain function audiovisual stimulation system (Mead SA-9927) compatible with the MRI interface for presentation.

### 2.3. Procedures

The auditory stimuli were played to the participants via MRI-compatible headphones connected to a computer. The stimulus intensity was set to 75 dB and kept constant during the process. The cue for the stimulus task (cross) was projected with a projector onto a screen behind the aperture of the MRI device during the scan. The participants could see the cues via a reflector mounted on the head coil. After hearing the stimuli, they were asked to provide feedback on the task via the keyboard. Before starting the experiment, a training session was provided to the participants to familiarize them with the procedure.

**Figure 2:**
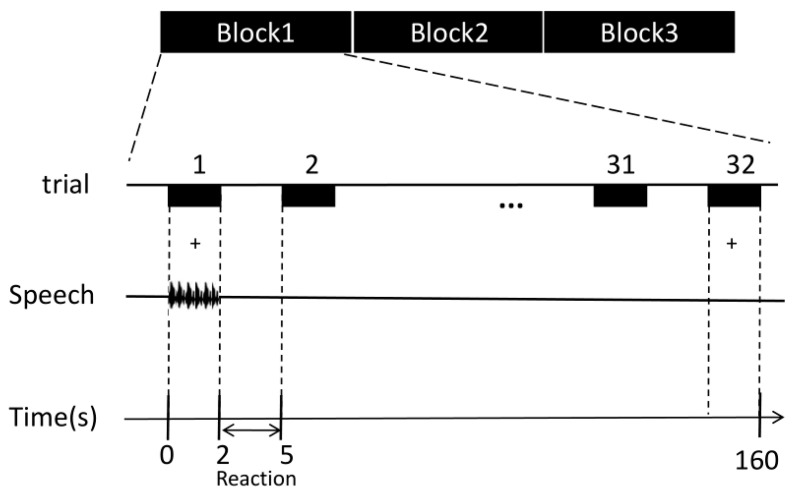
Diagram of the speech stimulation paradigm. Experiments were designed in randomized blocks: glottal stop, hypernasal speech, and typical speech. Each block contained 32 speech stimuli (randomly presented). Each trial lasted for 2 s, and the response time was 3 s.

A block design was used in the experiment (Figure 2). Three blocks of speech stimuli were presented in random order. Thirty-two speech stimuli were presented at once every 5 s in each block. For each trial, the speech stimulus presentation lasted for 2 s, and the participant’s response lasted for 3 s. The participants were instructed to passively listen to the phrases with the headphones while giving feedback regarding the perception of the stimuli. The behavioral responses of the participants were recorded simultaneously using E-prime 3.0 software.

### 2.4. Task-fMRI Imaging

The scanning task was performed at the Department of Radiology, Jiangsu Province Hospital of Chinese Medicine, Affiliated Hospital of the Nanjing University of Chinese Medicine. The scan was conducted using a 3.0T MRI scanner (GE Signa architect). The participants were placed in a supine position. The space between the head and outer coil was filled with a sponge pad to reduce head movement. A senior professional technologist performed all scanning procedures.

A gradient echo sequence was initially used to acquire high-resolution T1 anatomical images to align with functional data during preprocessing. Patients with structural brain abnormalities were excluded. The scan parameters were as follows: repeat time (TR) = 9.4 ms, echo time (TE) = 4.6 ms, flip angle (FA) = 10°, field of view (FOV) = 240 × 240 mm^2^, continuous scan, 180 layers, and layer thickness = 1 mm. Functional images were subsequently scanned using a gradient echo sequence with the following parameters: TR = 2000 ms, TE = 30 ms, FOV = 224 × 224, acquisition matrix = 64 × 64 × 16, voxel size = 3.75 × 3.75 × 3, and FA = 90°. The number of scans was 33 layers, with a thickness of 3.5 mm for each layer and no layer gap.

### 2.5. fMRI Data Analysis

Preprocessing and statistical analysis were performed using the functional neuroimaging analysis software SPM12 (version 6225, https://www.fil.ion.ucl.ac.uk/spm/, accessed on 9 October 2021). Preprocessing included the following steps: Initially, the first three volumes were removed to prevent artifacts from the initial signal instability of fMRI. Next, head motion correction was performed. The scans were aligned to the first image. Then, the structural image was coregistered with the mean functional image. The parameters from the segmentation of the structural image were used to normalize the functional images that were resampled to 3 × 3 × 3 mm. The realigned normalized images were then smoothed with a Gaussian kernel with a 6 mm full-width half-maximum to conform to random field theory assumptions and improve the sensitivity of group analysis.

A general linear model was used in the individual-level analysis with SPM12 for each of the three speech stimulus conditions. A statistical matrix involving the stimulus task with a canonical hemodynamic response function was created. The obtained individual-level data were subsequently used for group analysis at the second level. Bonferroni correction, with a *p* value of <0.05, was applied for second-level analysis with a clump threshold of 20 voxels. The different brain activation patterns were statistically analyzed when perceiving hypernasal speech, the glottal stop, and typical speech.

## 3. Results

Using within-subject analysis of variance, the brain activation patterns of the glottal stop, hypernasal speech, and typical speech were compared. Results showed that there was a significant difference among the following brain areas: the right fusiform gyrus, right lingual gyrus, left orbital part and triangular part of inferior frontal gyrus, left inferior occipital gyrus, left superior temporal gyrus, left medial superior frontal gyrus, right angular gyrus, and left inferior parietal gyrus.

### 3.1. Perception of the Glottal Stop in Typical Adult Listeners

**Figure 3:**
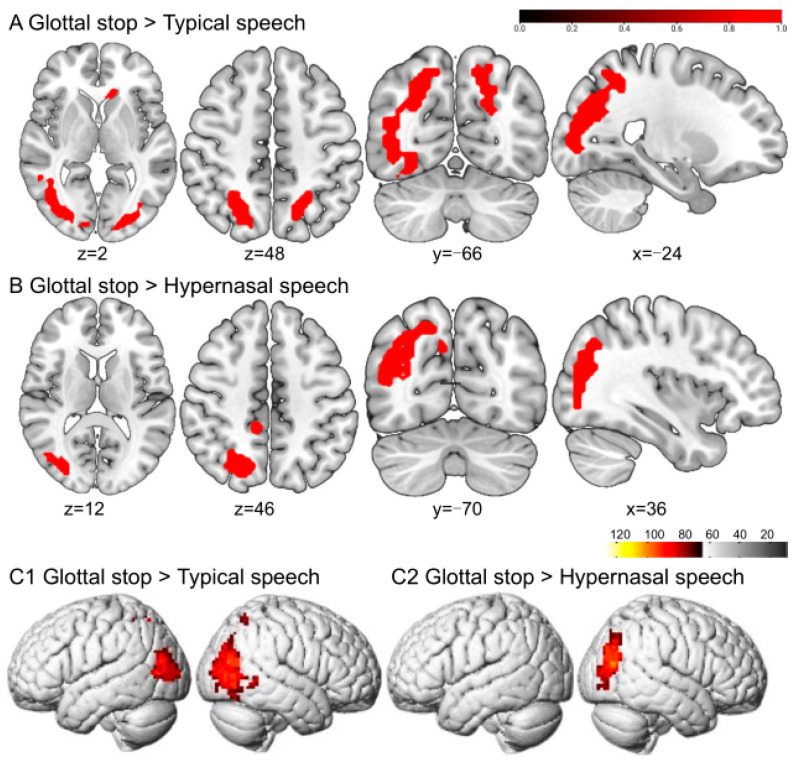
The perception of the glottal stop in typical adult listeners. (**A**,**B**) show the sliced view, and (**C**) depicts the rendered view. The warm-colored areas in (**A**) indicate glottal stop > typical speech, regions of difference: right FFG and left IOG. The warm-colored areas in (**B**) show glottal stop > hypernasal speech, regions of difference: right FFG. (**C1**) depicts the stimulation pattern of glottal stop > typical speech. (**C2**) shows the stimulation pattern of glottal stop > hypernasal speech (Bonferroni corrected, *p* < 0.05).

Greater BOLD responses to the glottal stop than to typical speech conditions were localized in the right fusiform gyrus (FFG) and left inferior occipital gyrus (IOG). The regions responding to the glottal stop rather than to hypernasal speech were located in the FFG (Figure 3 and Table 1).

**Table 1:** Peak voxels of significant activation clusters among the three different auditory stimuli: cleft-related hypernasal speech, cleft-related glottal stop, and typical speech.

		Coordinates (MNI)				
Regions (AAL)	Approx. BA	x	y	z	Peak t Value	Cluster Size (Voxel)
Glottal > Typical						
R FFG	19	39	−66	−18	9.35	939
L IOG	40	−24	−87	−3	9.24	568
Glottal > Hypernasal						
R FFG	19	27	−87	−6	8.96	513
Hypernasal > Glottal						
L ORBinf	47	−45	30	−9	6.98	28
L MTG	21	−57	-24	−3	6.72	20
Typical > Glottal						
L ITG	20	−48	−6	−33	7.59	20
L STG	38	−42	0	−9	8.43	27
L SFGmed	9	0	51	24	7.13	27
R ANG	39	54	−66	30	11.73	58

The paired *t*-test was used between any two auditory stimuli with a clumping threshold of 20 voxels. FFG: fusiform gyrus; IOG: inferior occipital gyrus; ORBinf: orbital part of the inferior frontal gyrus; MTG: middle temporal gyrus; ITG: inferior temporal gyrus; STG: superior temporal gyrus; SFGmed: medial superior frontal gyrus; ANG: angular gyrus; L: left; R: right. Atlas labeling was performed according to the AAL atlas. BA = Brodmann area.

### 3.2. Perception of Hypernasal Speech in Typical Adult Listeners

**Figure 4:**
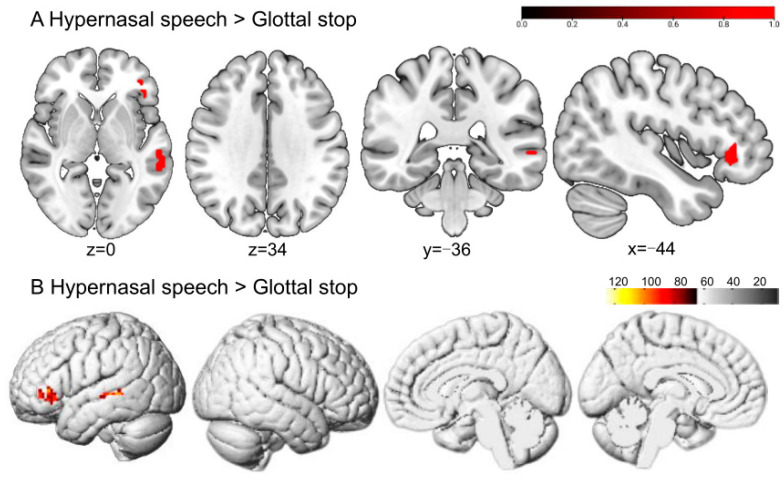
The perception of hypernasal speech in typical adult listeners. (**A**,**B**) show the sliced and rendered views, respectively. The warm-colored areas in (**A**) indicate hypernasal speech > glottal stop, regions of difference: left ORBinf and MTG. (**B**) depicts the stimulation pattern of hypernasal speech > glottal stop (Bonferroni corrected, *p* < 0.05).

Greater BOLD responses to hypernasal speech than to the glottal stop were localized in the left orbital part of the inferior frontal gyrus (ORBinf) and middle temporal gyrus (MTG), without significant activation differences in typical speech (Figure 4 and Table 1).

### 3.3. Perception of Typical Speech in Typical Adult Listeners

**Figure 5:**
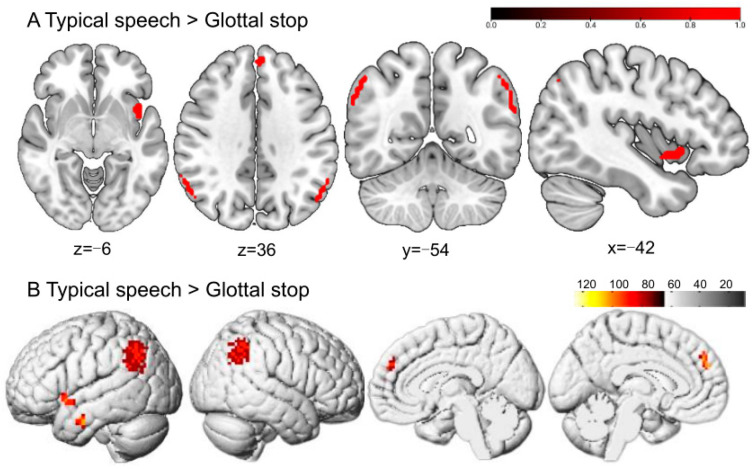
The perception of typical speech in typical adult listeners. (**A**,**B**) show the sliced and rendered views, respectively. The warm-colored areas in (**A**) indicate typical speech > glottal stop, regions of difference: left ITG, left STG, left SFGmed, and right ANG. (**B**) depicts the stimulation pattern of typical speech > glottal stop (Bonferroni corrected, *p* < 0.05).

Greater BOLD responses to typical speech than to the glottal stop were localized in the left inferior temporal gyrus (ITG), left superior temporal gyrus (STG), left medial superior frontal gyrus (SFGmed), and right angular gyrus (ANG). No significant difference was reported in typical speech when compared with hypernasal speech (Figure 5 and Table 1).

## 4. Discussion

This study aimed to explore the different brain activation patterns when perceiving two speech samples, namely the glottal stop and hypernasal speech, in a group of typical adult listeners through task-fMRI. Results revealed that compared with typical speech and hypernasal speech, the glottal stop, a typical representation of ACPS, could activate brain regions in the right FFG and the left IOG. Hypernasal speech, a typical representation of PCPS, could activate the left ORBinf and left MTG, compared with the glottal stop. Simultaneously, typical speech produced more significant activation responses in several brain regions, including the left ITG, left STG, left SFGmed, and right ANG, compared with the glottal stop. The results suggested that typical adult listeners may use different neural processing to perceive cleft-related speech produced by patients with cleft palate. This would provide a vital reference for the perceptual pattern of cleft-related speech signals. Further, the dual-stream model may be applied to cleft-related speech.

### 4.1. Analysis of Brain Activation Pattern for the Perception of the Glottal Stop

The glottal stop is a cleft-related speech pattern produced with the confliction of the vocal folds. This misarticulation is often used as a substitution for different oral consonants. For example, in pronouncing “/ba ba/” (father in Chinese), the bilabial /b/ may be produced as “/ʔa ʔa/”. The current study found that perceiving the glottal stop did not activate the ventral or dorsal stream in the classic pathway for speech perception among typical adult listeners. Instead, it activated the right FFG and left IOG. Previous studies have revealed that activation increased in the bilateral FFG while processing non-native reading tasks [20]. The right FFG was also involved in facial recognition tasks [21]. Thus, it is reasonable to speculate that the increased activation of the FFG was caused by a high cognitive response when processing “unfamiliar signals” in the brain. A previous study reported that the IOG was involved in the visual and mental imaging process [22]. The activation of the visual network in the right IOG identified while perceiving the glottal stop was also reported in high-cognitive activities, such as visual and mental imagery to improve speech recognition. It could also be related to typical listeners’ visual imagination to localize a strange articulatory place [23]. Vetter observed that natural sounds and some other auditory signals (such as forest sounds, crowd sounds, and vehicle sounds) were decoded in the primary visual cortex [24]. Thus, the glottal stop carries information that must be decoded by the visual cortex.

There may be two possible reasons that the glottal stop could not effectively activate the classical speech perception pathway. First, the glottal stop is not in the Mandarin Chinese phonological system (while it could be found in some dialects in Chinese). When typical speakers use their native language, the STG is triggered, which is responsible for the automatic processing of lexical retrieval and constructing phrases. In contrast, the activation intensity of the STG could decrease when using a second foreign language compared with the native language. A bilingual user with a lower skill in the second language could recall other cognition-related brain regions, such as the IFG, to decode the second language [25]. If proficiency in the second language improves, the bilingual user’s brain activation pattern during native and foreign language usage could become similar [26]. The abnormal pathway of perceiving the glottal stop could also validate a significant difference in the typical speech system.

Second, it may be related to the low intelligibility of the glottal stop. Brain neuroscience studies have shown that clear speech activates the speech cortex, including the STG and superior temporal sulcus (STS) [27,28]. In contrast, unclear speech could not activate such a brain activation response. A series of fMRI studies with univariate analysis found that clear speech produced more pronounced brain activation responses in regions such as the left superior temporal area, inferior parietal area, and IFG. A whole-brain multifactor analysis based on fMRI revealed that clear speech activated a broader range of brain networks, including the left MTG, ANG, inferior temporal cortex, and IFGtriang. Functional brain connectivity was also observed to be more strongly activated by clear speech [29]. Functional near-infrared spectroscopic imaging revealed that activation responses in superior temporal regions varied linearly with speech intelligibility. By contrast, the left inferior frontal cortex, bilateral inferior parietal cortex, and middle temporal cortex had a nonlinear activation pattern with the index of speech intelligibility [30].

Instead of the common articulation produced in the oral cavity, the glottal stop is produced at a relatively lower level of articulation, which leads to poor intelligibility. An acoustic analysis of the glottal stop could provide evidence for this. The spectrum of the glottal stop has a different distribution than the oral stop. The major energy of the glottal stop is in a low-frequency region. In a high-frequency area, the power of the glottal stop is lower than that of the oral sound. Previous studies on the pharyngeal fricative, another ACPS, showed that a spectral energy < 4 kHz was relatively higher than those of typical fricatives. In contrast, a spectral power > 4 kHz was lower, with two spectral energy peaks at approximately 800 and 2.5 kHz, respectively [31]. Simultaneously, if patients extensively substitute oral consonants with the glottal stop, the distinctive feature between the minimal phoneme pair is lost. Thus, it could worsen the intelligibility further.

However, behavioral studies revealed that ACPS was easier to perceive than PCPS for parents of children with repaired CLP [32]. This is contradictory to the results of the current study, where typical adult listeners could not perceive ACPS well. The difference may be attributed to the difference between the participants. In our study, only typical adult listeners without cleft palate speech exposure have been investigated. These typical listeners were not familiar with the phonological system of ACPS. Thus, it was perceived as an unknown speech sound. In contrast, the parents of children with ACPS may perceive it as clearer, as familiarity plays an essential role.

### 4.2. Analysis of Brain Activation Pattern for the Perception of Hypernasal Speech and Typical Speech

Hypernasal speech is caused by a cleft palate or velopharyngeal insufficiency. It results in excessive nasal resonance due to incomplete separation between the oral and nasal cavities when pronouncing oral phonemes. It could mainly influence the pronunciation of vowels and some high-pressure consonants. The current experiment showed that hypernasal speech, compared with the glottal stop, increased activation in brain regions involving the left ORBinf and the left MTG among typical listeners. More significant activation responses to typical speech were produced in several brain regions, including the left ITG, left STG, left SFGmed, and right ANG, compared with the glottal stop. Further, there was no significant perceptual difference between typical speech and hypernasal speech among typical listeners.

The temporal and frontal lobes identified in the mentioned study were in accordance with the dual-stream model [33,34]. This finding showed that cleft-related hypernasal speech could activate the dual-stream model of classical speech perception in typical listeners. The dual-stream model may be applicable to cleft-related speech to a certain extent. Moreover, it is reasonable to speculate that the brain may perceive hypernasal speech similarly to typical speech, and the neural correlates of speech perception could be similar.

### 4.3. Limitations and Future Research

Although the current study revealed some important features of perceiving cleft-related speech, there were still some limitations. First, the auditory stimuli of cleft-related speech used in this study were collected from three participants. The corpus should be more widely representative. Future studies must use more comprehensive corpora of auditory speech, including various hypernasality levels and different compensatory misarticulation types. Second, further studies must investigate the perception features of different types of adults and children with NSCLP. Moreover, the current study did not explain why ACPS could not activate the perceptual pathway like typical speech. Hence, studies on the correlation between speech acoustics and brain activation responses must be conducted to explore the mechanism of the activation pattern. Further brain network analysis approaches, such as the dynamic causal model and brain network, can be used in the future to explore neural circuits for speech signal processing.

## 5. Conclusions

Using the task-fMRI technique, the current study investigated the perceptual pattern of cleft-related speech, including the glottal stop, hypernasal speech, and typical speech, among typical adult listeners. The results showed that the glottal stop, a typical pattern of ACPS, could activate the right FFG and left IOG. In contrast, hypernasal speech, a typical pattern of PCPS, could activate the left ORBinf and MTG. In addition, typical speech was significantly activated in several brain regions, including the left ITG, left STG, left SFGmed, and right ANG. The typical adult listener could initiate different neural processing to perceive cleft-related speech. The result could also provide some preliminary data for exploring the application of the dual-stream model in cleft-related speech. It is highly recommended that further investigation be conducted to explore the relationship between acoustic parameters and the activation pattern of perceiving cleft-related speech. This could uncover the mechanism of a distinctive neural pathway for cleft-related speech.

## Data Availability

The data that support the findings of this study are available from the corresponding author upon reasonable request.

## Supplementary Materials

The following supporting information can be downloaded at https://www.mdpi.com/article/10.3390/brainsci13111506/s1, Table S1: Content of the auditory speech stimuli, and Table S2: Sociodemographic characteristics of the participants.
