# Supplementary material for: Perceptual Pattern of Cleft-Related Speech: A Task-fMRI Study on Typical Mandarin-Speaking Adults
Source: Brain Sci. 2023 Oct 25;13(11):1506. doi: 10.3390/brainsci13111506 (PMC10669275; doi:10.3390/brainsci13111506)
Supplement: Supplementary file 1 [file brainsci-13-01506-s001.zip › Supplementary Table S1.pdf]

Supplementary S1: Content of the auditory speech stimuli

No.	Stimuli for glottal stop (in Chinese)	Pin Yin	Stimuli for hypernasal speech (in Chinese)	Pin Yin	Stimuli for typical speech （in Chinese)	Pin Yin
1	是鼻子 nose	/shì bí zǐ/	三四 three and four	/sān sì/	这是爸爸 father	/zhè shì bà bà/
2	布鞋 shoes	/bù xié/	气 qi	/qì/	菠萝 pipeapple	/bō luó/
3	菠萝 pipeapple	/bō luó/	九十 ninety	/jiǔ shí/	葡萄 grape	/pú táo/
4	是胡萝卜 carrots	/shì hú luó bo/	布鞋 shoes	/bù xié/	这妈妈 mother	/zhè mā mā/
5	葡萄 grape	/pú táo/	机器人 robot	/jī qì rén/	这小孩 kids	/zhè xiǎo hái/
6	蜜蜂 honeybee	/mì fēng/	猪八戒 porky pig	zhū bā jiè	这是木头 wood	/zhè shì mù tóu/
7	木头 wood	/mù tóu/	刺猬 hedgehogs	/cì wei/	这西瓜 watermelon	/zhè xī guā/
8	小蘑菇 mushroom	/xiǎo mó gū/	醋 vinegar	/cù/	这是飞机 aircraft	/zhè shì fēi jī/
9	这西瓜 watermelon	/zhè xī guā/	厨师 chef	/chú shī/	这是什么呀 shenme	/zhè shì shén me ya/
10	斧头 axe	/fǔ tóu/	爬楼梯 stair	/pá lóu tī/	大象 elephant	/dà xiàng/
11	佛像 buddha	/fó xiàng/	琵琶 lute	/pí pá/	汽车 car	/qì chē/
12	大象 elephant	/dà xiàng/	葡萄 grape	/pú táo/	踢球 kick	/tī qiú/

13	弟弟 brother	/dì dì/	婆婆 grandmother	/pó pó/	这两只小鸟 birds	/zhè liǎng zhī xiǎo niǎo/
14	肚皮 belly	/dù pí/	发夹 hair clip	/fā jiá	喇叭 horn	/lǎ bā/
15	抬 lift	/tái/	飞机 aircraft	/fēi jī/	这是梨子 pear	/zhè shì lí zǐ/
16	在踢球 kick	/zài tī qiú/	佛 buddha	/fó/	嘎嘎 gaga	/gā gā/
17	小兔子 rabbit	/xiǎo tùzǐ/	大象 elephant	/dà xiàng/	打鼓 drum	/dǎ gǔ/
18	喇叭 horn	/lǎ bā/	弟弟 brother	/dì dì/	这卡车 truck	/zhè kǎ chē/
19	梨子 pears	/lí zǐ/	喇叭 horn	/lǎ bā/	可乐 coke	/kě lè/
20	嘎嘎 gaga	/gā gā/	蝴蝶 butterfly	/hú dié/	裤子 pant	/kù zǐ/
21	哥哥 brother	/gē gē/	红色 red	/hóng sè/	看电视机 TV	/kàn diàn shì jī/
22	手枪 pistols	/shǒu qiāng/	夹子 clip	/jiá zǐ/	这是哈密瓜 cantaloupe	/zhè shì hā mì guā/
23	是汽车 car	/shì qì chē/	菊花 chrysanthemum	/jú huā/	蝴蝶 butterfly	/hú dié/
24	裤子 pant	/kù zǐ/	酒杯 glasses	/jiǔ bēi/	这个气球 balloon	/zhè gè qì qiú/
25	我们家有电视 TV	/wǒmen jiā yǒu diàn shì/	去学校 school	/qù xué xiào/	这是枪 gun	/zhè shì qiāng/
26	哈密瓜 cantaloupe	/hā mì guā/	杂技 acrobatic	/zá jì/	这是皮球 ball	/zhè shì pí qiú/
27	蝴蝶 butterfly	/hú dié/	紫色 purple	/zǐ sè/	是蓝色 blue	/shì lán sè/
28	是苹果 apple	/shì píng guǒ/	足球 football	/zú qiú/	洒水 sprinkler	/sǎ shuǐ/
29	机器人 robot	/jīqìrén/	擦手 wipe	/cā shǒu/	猪八戒 porky pig	/zhū bā jiè/

30	去卫生间小便 toilet	/qù wèi shēng jian xiǎo biàn/	厕所 toilet	/cè suǒ/	他在吃蛋糕 cake	/tā zài chī dàn gāo/
31	是秋千 swing	/shì qiū qiān/	洒水 sprinkler	/sǎ shuǐ/	叉子 fork	/chā zǐ/
32	气球 balloon	/qì qiú/	四个 four	/sì gè/	滚轮胎的 tyre	/gǔn lún tāi de/
