# Supplementary material for: Perceptual Pattern of Cleft-Related Speech: A Task-fMRI Study on Typical Mandarin-Speaking Adults
Source: Brain Sci. 2023 Oct 25;13(11):1506. doi: 10.3390/brainsci13111506 (PMC10669275; doi:10.3390/brainsci13111506)
Supplement: Supplementary file 1 [file brainsci-13-01506-s001.zip › Supplementary Table S2.pdf]

Table S2: Sociodemographic characteristics of the participants

Subject	Gender	Age	WAIS Score
1	Female	22	116
2	Female	24	123
3	Female	28	107
4	Female	21	128
5	Female	18	97
6	Female	20	109
7	Female	25	121
8	Female	24	117
9	Female	26	123
10	Female	20	123
11	Female	22	121
12	Female	24	125
13	Female	28	112
14	Female	23	106
15	Female	24	115
16	Male	23	124
17	Male	20	127
18	Male	20	132
19	Male	24	124
20	Male	24	127
21	Male	20	103
22	Male	23	122
23	Male	26	110

24	Male	26	118
25	Male	24	124
26	Male	23	114
27	Male	22	129
28	Male	23	116
29	Male	24	121
30	Male	22	133

WAIS=Wechsler Adult Intelligence Scale
